# Dapagliflozin as an autophagic enhancer via LKB1/AMPK/SIRT1 pathway in ovariectomized/d-galactose Alzheimer’s rat model

**DOI:** 10.1007/s10787-022-00973-5

**Published:** 2022-04-01

**Authors:** Weam W. Ibrahim, Ahmed S. Kamel, Ahmed Wahid, Noha F. Abdelkader

**Affiliations:** 1 Department of Pharmacology and Toxicology, Faculty of Pharmacy, Cairo University, Kasr El-Aini St., Cairo, 11562 Egypt; 2 Department of Pharmaceutical Biochemistry, Faculty of Pharmacy, Alexandria University, Alexandria, Egypt

**Keywords:** Alzheimer’s disease, Autophagy, Ovariectomized/d-galactose, LKB1/AMPK signaling, Dapagliflozin

## Abstract

Autophagy and mitochondrial deficits are characteristics of early phase of Alzheimer’s disease (AD). Sodium-glucose cotransporter-2 inhibitors have been nominated as a promising class against AD hallmarks. However, there are no available data yet to discuss the impact of gliflozins on autophagic pathways in AD. Peripherally, dapagliflozin’s (DAPA) effect is mostly owed to autophagic signals. Thus, the goal of this study is to screen the power of DAPA centrally on LKB1/AMPK/SIRT1/mTOR signaling in the ovariectomized/d-galactose (OVX/d-Gal) rat model. Animals were arbitrarily distributed between 5 groups; the first group undergone sham operation, while remaining groups undergone OVX followed by d-Gal (150 mg/kg/day; i.p.) for 70 days. After 6 weeks, the third, fourth, and fifth groups received DAPA (1 mg/kg/day; p.o.); concomitantly with the AMPK inhibitor dorsomorphin (DORSO, 25 µg/rat, i.v.) in the fourth group and the SIRT1 inhibitor EX-527 (10 µg/rat, i.v.) in the fifth group. DAPA mitigated cognitive deficits of OVX/d-Gal rats, as mirrored in neurobehavioral task with hippocampal histopathological examination and immunohistochemical aggregates of p-Tau. The neuroprotective effect of DAPA was manifested by elevation of energy sensors; AMP/ATP ratio and LKB1/AMPK protein expressions along with autophagic markers; SIRT1, Beclin1, and LC3B expressions. Downstream the latter, DAPA boosted mTOR and mitochondrial function; TFAM, in contrary lessened BACE1. Herein, DORSO or EX-527 co-administration prohibited DAPA’s actions where DORSO elucidated DAPA’s direct effect on LKB1 while EX-527 mirrored its indirect effect on SIRT1. Therefore, DAPA implied its anti-AD effect, at least in part, via boosting hippocampal LKB1/AMPK/SIRT1/mTOR signaling in OVX/d-Gal rat model.

**Graphical abstract:**

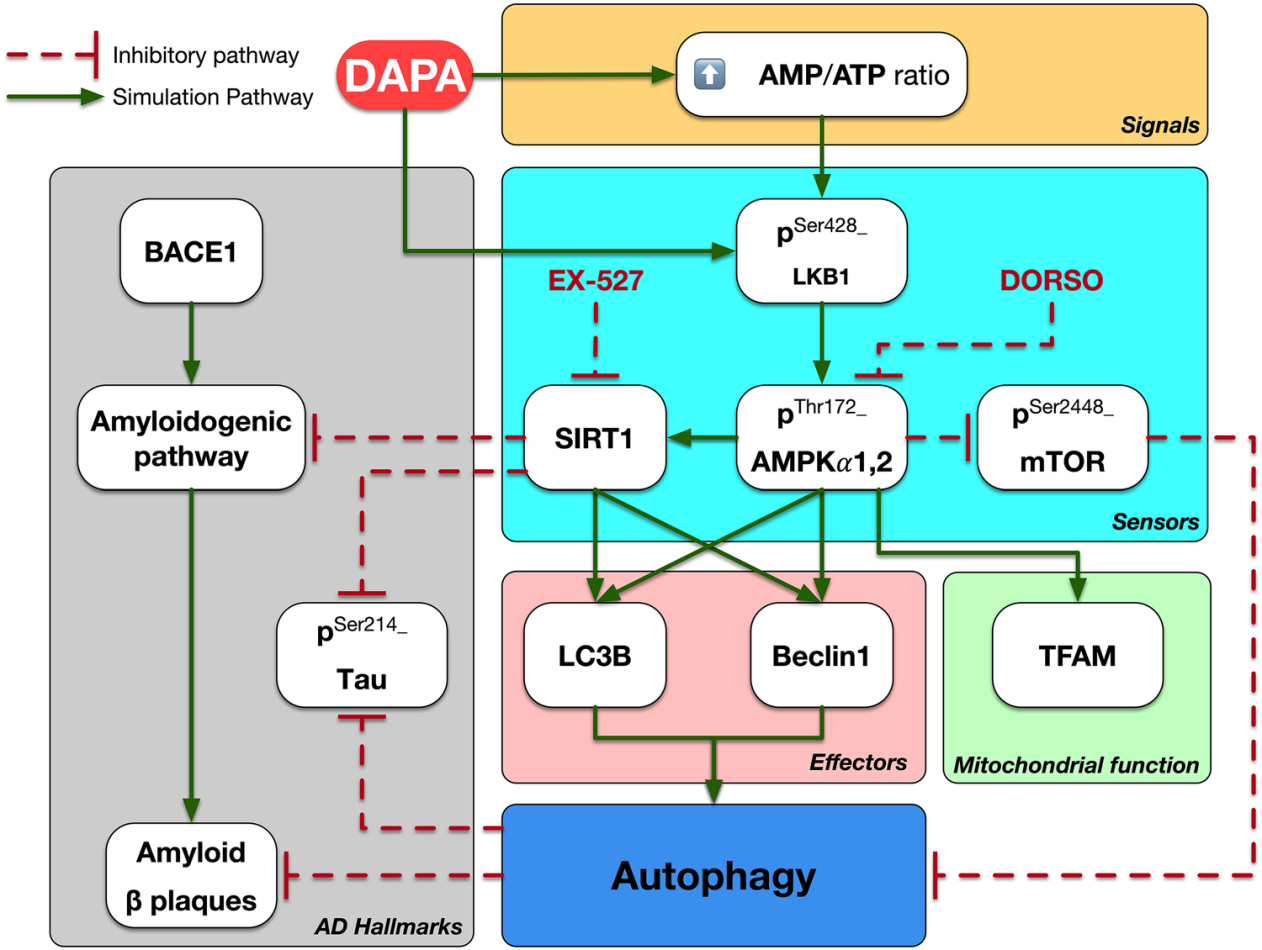

## Introduction

Alzheimer’s disease (AD) is a very common form of dementia that affects more than 50 million people around the world, especially the elderly people. Cognitive impairment, memory loss, and behavioral disturbances are all characteristics of AD. The prime neuropathological features of the disease in the brain include extracellular deposits of amyloid-β (Aβ) in senile plaques as well as abnormal accumulation of phosphorylated Tau (p-Tau) protein intra-neuronally forming neurofibrillary tangles, Unfortunately, the detailed mechanism underlying AD etiology and pathogenesis has not been yet fully elucidated (Durairajan et al. 2021).

Autophagy is a normal degradation process, which via lysosomal degradation clears unnecessary or dysfunctional components such as aggregated protein species and damaged organelles, thus promoting cellular fitness. Changes in autophagy and lysosomal function are clear markers for neurodegenerative diseases (Bastien et al. 2021). It has been previously proposed that autophagy is involved in the elimination of many toxic aggregates. Indeed, Aβ is cleared by microglia via autophagy; however, this process is stopped in AD patients (Estfanous et al. 2021).

Autophagy relies on a machinery organized by several key energy sensors such as adenosine monophosphate-activated protein kinase (AMPK) and mechanistic target of rapamycin (mTOR). AMPK plays a crucial role in cellular energy homeostasis. It is a master metabolic regulator that is sensitive to any alterations in energy status (Lin and Hardie 2018). When adenosine triphosphate (ATP) is not effectively generated by glycolysis or oxidative phosphorylation, the energy charge declines with a concomitant AMP accumulation, a condition that activates autophagy via AMPK (Hardie et al. 2012). AMPK activation is a key metabolic regulator of the autophagy pathway including microtubule-associated protein light chain 3 (LC3B) and Beclin1. Whereas mTOR was previously reported to possess an inhibitory role in the autophagy pathway (Ha et al. 2015; Garcia and Shaw 2017). Furthermore, AMPK can promote autophagy via mTOR inhibition under conditions of reduced ATP levels (Alers et al. 2012). Chief among these findings, AMPK and AD share promising interactive pathways. Liver kinase B1(LKB1)/AMPK pathway, one of the main metabolic kinases, has a crucial role in AD progression as a neuroprotective signaling. In context, AMPK mitigates Tau phosphorylation by decreasing the activity of glycogen synthase kinase-3β, the main Tau kinase. Moreover, sirtuin 1 (SIRT1), a major downstream molecule in AMPK signaling, enhances the deacetylation and degradation of misfolded Tau (Assefa et al. 2020).

Previous preclinical and clinical studies have explored the possible neuroprotective mechanisms of different classes of antidiabetic drugs through autophagic machinery in AD, with promising results (Ashrafizadeh et al. 2019). One of the newest classes of oral antidiabetics is sodium-glucose cotransporter-2 (SGLT2) inhibitors. Existence of SGLT2 in the mammalian central nervous system, specifically in the hippocampus, cerebellum, and blood−brain barrier (Wiciński et al. 2020), has directed the research towards investigating the neuroprotective potential of SGLT2 inhibitors. Interestingly, their neuroprotective benefits have been recently reported experimentally in stroke, Huntington’s disease, and Parkinson’s disease, regardless of their glucose-lowering ability (Abdel-latif et al. 2020; El-Sahar et al. 2020; Arab et al. 2021). Noteworthy, dapagliflozin (DAPA), a novel SGLT2 inhibitor, was reported to revert autophagic flux reduction (Xu et al. 2021). In agreement with this, a recent investigation indicated DAPA’s capacity to activate AMPK phosphorylation and inhibit mTOR phosphorylation both in vitro and in vivo (Luo et al. 2021). In addition, it has been shown that DAPA restored impaired autophagy in high glucose-treated HK-2 cells and in high-fat diet-induced obesity in rats (Xu et al. 2021; Jaikumkao et al. 2021). Additionally, DAPA was reported to attenuate motor dysfunction and neuronal injury in rat models of Huntington’s and Parkinson’s diseases via suppressing NF-κB-mediated inflammation, as well as mitigating oxidative stress, mitochondrial dysfunction, and apoptosis (El-Sahar et al. 2020; Arab et al. 2021). Furthermore, DAPA may exhibit acetylcholinesterase inhibiting activity, which makes it a good candidate for treating AD (Wiciński et al. 2020; Shaikh et al. 2014).

Of note, further investigation into the mechanism by which DAPA activates LKB1 is still needed. Specifically, it was previously suggested that empagliflozin, another SGLT2 inhibitor, might activate LKB1 either directly by phosphorylation or through SIRT1 (Lu et al. 2020). SIRT1 can activate LKB1 by increasing its deacetylation and consequently its phosphorylation (Wang et al. 2018).

This unprecedented study was set out to learn more about the role of SGLT2 inhibition and its influence on autophagic machinery in AD. Administration of d-galactose (d-Gal) for a long period to ovariectomized (OVX) rats results in behavioral, neurochemical, and pathological alterations similar to AD (Hua et al. 2007; Ibrahim et al. 2019, 2020; Kamel et al. 2018). Thus, the OVX/d-Gal rat model was used in the current study with the aim of screening the power of DAPA centrally on LKB1/AMPK/SIRT1/mTOR signaling in AD. The AMPK and SIRT1 inhibitors; dorsomorphin (DORSO) and EX-527, respectively, were also used herein to study the level of DAPA involvement in autophagic pathway.

## Materials and methods

### Animals

Female Wistar rats (3–4 months old, 160 ± 20 g), were acquired from the animal facility of Faculty of Pharmacy, Cairo University (Cairo, Egypt). Rats were housed in this facility under proper conditions in terms of temperature (23 ± 2 °C), humidity (60 ± 10%), and light/dark cycle (12/12 h, lights on at 7:00 am). Rats were given chow diet and water ad libitum. All efforts were exerted to decrease the suffering of animal throughout the investigational period.

### Compliance with ethical standards

The experimental protocol (permit number: PT-3047) is consistent with the Guide for the Care and Use of Laboratory Animals protocol (NIH publication No. 85–23, revised 2011) instigated by the Ethics Research Committee of Faculty of Pharmacy, Cairo University.

### Drugs and chemicals

d-Galactose, DAPA, DORSO, and EX-527 were acquired from Sigma-Aldrich Chemical Co. (Missouri, USA). Physiological saline was used for the preparation of d-Gal and DAPA, while DORSO and EX-527 were dissolved using dimethyl sulfoxide (Merck, Germany) and freshly diluted with physiological saline. Other chemicals were of the highest analytical grade.

### Ovariectomy

Rats have been anesthetized using ketamine and xylazine (50 and 10 mg/kg, i.p., respectively). On each lateral side of the abdomen, the area between the hip and the last rib was shaved and disinfected by 70% alcohol, afterwards a small notch was made in this region. After exteriorizing the ovaries and their associated oviducts, a hemostatic clamp was put directly under the ovaries to produce a suture knot. Afterwards, ovaries were cut with sterile scissors and discarded. Absorbable and nonabsorbable threads were used to stitch the muscle and skin layers, respectively. Sham operation was performed in the same way, except for ovaries removal. All rats received 0.1 ml cefotaxime (100 mg/ml) and 0.1 ml diclofenac sodium subcutaneously after the surgery. Animals were maintained on diet free of soy to rule out the effect of phytosteroids (Khajuria et al. 2012; Abdelkader et al. 2020).

### Experimental design

**Fig. 1:**
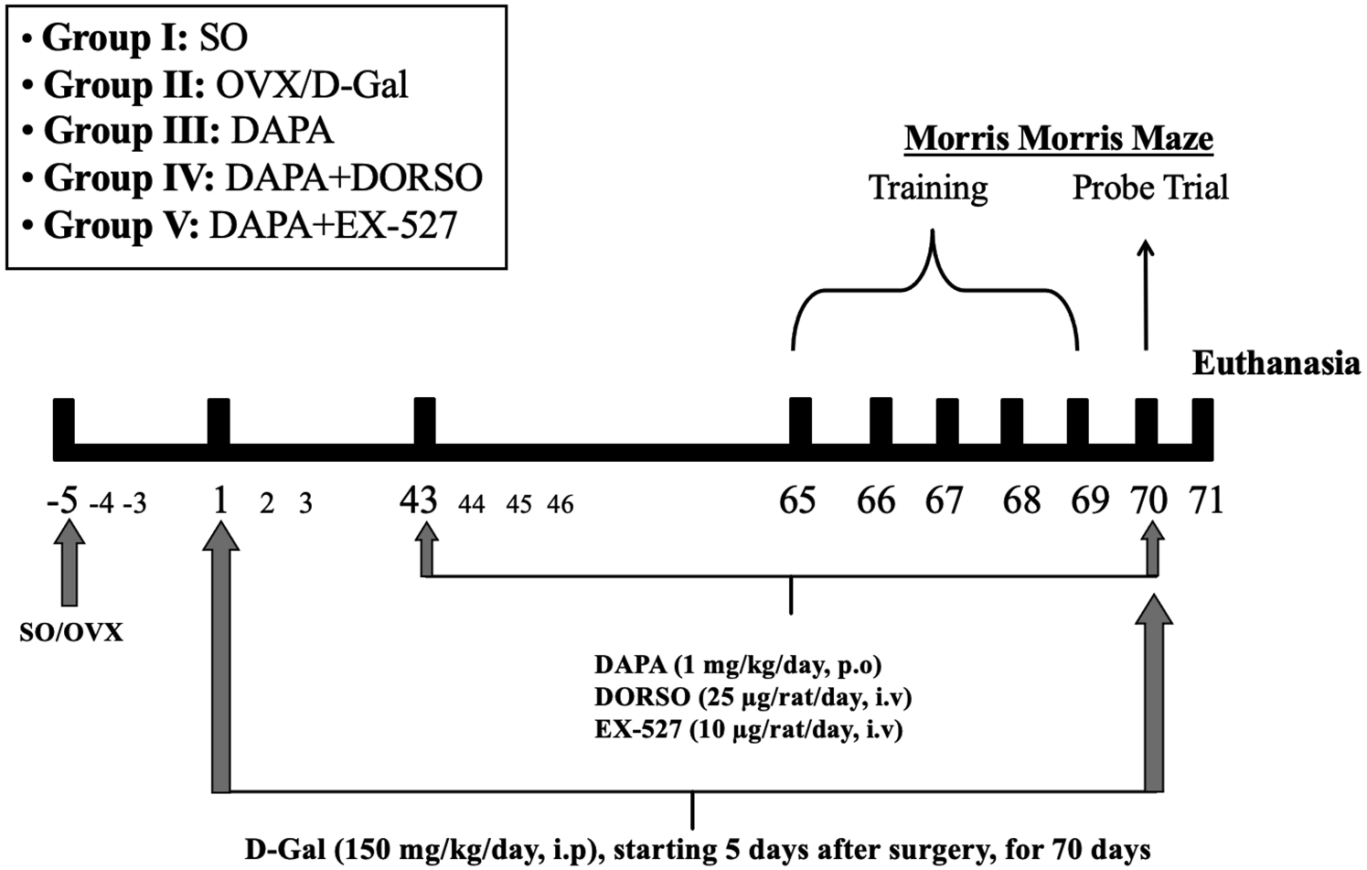
Experimental design

Rats were arbitrarily assigned into five groups, each containing 15 rats (Fig. 1). Group I (SO): sham-operated rats that served as control group. Group II (OVX/d-Gal): rats were subjected to bilateral OVX and received d-Gal (150 mg/kg/day, i.p.) for 70 days, starting 5 days after surgery (Ibrahim et al. 2020). Group III (DAPA): OVX/d-Gal rats that were treated orally with DAPA (1 mg/kg/day) (El-Sahar et al. 2020) for a period of 28 days, starting after 6 weeks of d-Gal administration (43rd day). Group IV (DAPA + DORSO) and group V (DAPA + EX-527): OVX/d-Gal rats treated with DAPA; as group III, and either treated concomitantly with DORSO (25 µg/day/rat, iv) (Safar et al. 2021) or EX-527 (10 µg/day/rat, iv) (Shi et al. 2021), which were given 15 min before DAPA for 28 days, starting after 6 weeks of d-Gal administration (43rd day). Five days before ending the experiment, rats have been exposed to the Morris water maze (MWM) behavioral test where the training phase was executed over 4 days (days 66–69) and the probe trial was conducted on the fifth day (day 70). The performance of animals in MWM test was monitored using ANY-Maze video tracking software (Stoelting Co, USA).

### Behavioral assessment

Spatial memory in rodents is assessed using MWM test. The maze consisted of a round pool (diameter: 150 cm, height: 60 cm), containing 40 cm deep water which was made opaque using a non-toxic water-soluble black paint. The pool was divided arbitrarily into four equal quadrants with a platform (diameter: 8 cm) located in the middle of a certain quadrant, just below the water surface. In the acquisition phase, 2 training sessions (120 s each) were given for each rat for a period of 4 days (days 66–69). In the training session, each rat was placed in the pool and left free to reach the platform in the target quadrant. If the rat reached the platform within 120 s, it was allowed to stay on it for 10 s, while, if it failed to reach the platform within the designated time, it was gently guided to the platform and placed on it for 30 s. On the fifth day (day 70), the probe test was conducted by removing the platform, then placing each rat in the quadrant opposite to the target quadrant and it was left to explore the pool for 60 s. Time spent swimming in the target quadrant, latency to enter target quadrant, number of target quadrant crossings, mean speed, and time spent swimming in the opposite quadrant were recorded using an overhead camera (Sayed et al. 2020).

### Brain processing

One day following MWM probe trial (day 71), rats were euthanized by decapitation under anesthesia, and the brains were quickly removed, washed with ice-cold saline, dried, and weighed. In each group, rats’ brains were allocated into three subsets. In the first subset (*n* = 3), brains were processed for the histological investigation and immunohistochemistry determination of p-Tau. To eliminate bias, all histopathological and immunohistochemical examinations were performed by an experienced investigator who was blinded to the identity of the samples. In the other two subsets, the hippocampi were dissected out from each brain and flash frozen in liquid nitrogen, then kept at − 80 °C for further biochemical investigations. Hippocampi from the second subset (*n* = 6) were homogenized in ice-cold phosphate-buffered saline to make a 10% (w/v) homogenate to estimate adenosine monophosphate (AMP), ATP, and β-site amyloid precursor protein cleaving enzyme 1 (BACE1) contents. The right hippocampi from the third subset (*n* = 6) were used for mitochondrial separation to assess transcription factor A mitochondrial (TFAM), while the left hippocampi of 3–4 rats were used to determine p-AMPKα1,2, LC3B, p-LKB1, SIRT1, p-mTOR, and Beclin1 relative expression using Western blot analysis.

### Histopathological examination

Brain specimens were fixed in neutral buffered formalin (10%) for 72 h. Then, they were treated with ethanol in serial grades, cleared in xylene, infiltrated, and finally embedded into Paraplast tissue embedding media (Leica biosystems, Wetzlar, Germany). Four micron thick serial coronal brain sections were cut, mounted on glass slides, and stained by hematoxylin and eosin (H&E) for microscopic examination of hippocampal subregions. In addition, Nissl staining analysis was performed by staining the coronal sections with toluidine blue (Culling 2013). Four random non-overlapping fields per section were analyzed for the quantification of intact neurons, showing intact subcellular details, in the CA3 region of the hippocampus. For histological analysis, all micrographs were taken with a full HD microscopic imaging system operated with Leica application module (Leica Microsystems GmBH, Wetzlar, Germany).

### Immunohistochemical examination

Paraffin-embedded tissue sections were deparaffinized followed by incubation with hydrogen peroxide (0.3%) for 20 min. Sections were then incubated with anti-p-Tau ^Ser214^ antibody (1:100, Thermo Fisher Scientific, IL, USA; catalogue number: 44-742G) overnight at 4 °C. The samples were rinsed with PBS and incubated for 20 min with secondary antibody HRP Envision kit (Dako, CA, USA). Following another PBS wash, sections were incubated for 15 min with 3, 3′-diaminobenzidine tetrahydrochloride (DAB Substrate Kit, Vector Laboratories Inc., CA, USA). They were rewashed with PBS, counterstained with hematoxylin, dehydrated, cleared in xylene, and cover slipped for light microscopic investigation. Quantification of p-Tau in pyramidal neurons was performed by measuring the optical density from four random fields in each section and averaged using the image analysis software (Image J, version 1.46a, NIH, MD, USA) (Abdelkader et al. 2022).

#### Mitochondrial isolation

Hippocampi were homogenized in 0.7 M Tris–HCl buffer (pH = 7.4; Sigma-Aldrich Co., USA) containing 0.25 M sucrose (El-Nasr Pharmaceutical Co., Cairo, Egypt) and centrifuged at 2500×*g* for 10 min at 4 °C to remove nuclei and unbroken cells. Following decanting the supernatant fluid, primary mitochondrial pellets were formed by centrifugation at 10,000×*g* for 10 min at 4 °C. The pellet was washed by gently resuspending in 0.5 ml Tris–sucrose buffer then recentrifuged and the supernatant fluid was decanted. This step was repeated three times to improve the degree of mitochondrial purity. The final mitochondrial pellet was resuspended in Tris–sucrose buffer (Ahmed et al. 2014). The fresh mitochondrial suspension was used for the estimation of TFAM.

### Biochemical measurement

Using rat specific ELISA kits, the levels of AMP, ATP (Mybiosource, CA, USA; catalogue number: MBS7230212 and MBS723034, respectively), BACE1 (Biomatik, DE, USA; catalogue number: EKF58924), and TFAM (Biomatik, DE, USA; catalogue number: EKC39795) were assessed according to the standard protocol. The protein content of tissue homogenates and mitochondrial fractions was estimated by the Lowry method (Lowry et al. 1994).

### Western blot analysis

The amount of protein expression in the present study was examined by western blot assay. According to manufacturer’s instructions, 50–100 mg of hippocampal tissue was homogenized using TriFast protein extraction kit (Peqlab, VWR company). About 30 μg proteins were loaded and electrophoresis was performed at 75 then 125 V through stacking gel during 2 h. The resultant proteins on SDS-PAGE were transferred to a Hybond™ nylon membrane (GE Healthcare) and incubated for 1 h at room temperature in a blocking solution. Membranes were incubated overnight at 4 °C with primary antibodies (Thermo Fisher Scientific, IL, USA): p-LKB1 ^SER428^ (1:1000, catalogue number: PA5-36858), p-AMPKα-1,2 ^Thr172^ (1:700, catalogue number: PA5-37821), SIRT1 (1 μg/ml, catalogue number: PA5-20964), p-mTOR ^Ser2448^ (1:1000, catalogue number: 44-1125G), Beclin1 (0.5–2 μg/ml, catalogue number: PA5-20172), and LC3B (0.5–2 μg/ml, catalogue number: PA1-16930). Membranes were washed at room temperature for 30–60 min with continuous change of blotting buffer. Membranes were then incubated for 1 h at room temperature in a diluted HRP-conjugated secondary antibody (0.1–0.5 µg/ml) then the washing cycle was repeated. β-Actin was demonstrated as the housekeeping protein. A gel documentation system (Geldoc-it, UVP, UK) was applied for data analysis using Totallab analysis software (ww.totallab.com, Version 1.0.1).

### Statistical analysis

Results were expressed as mean ± SD. Statistics was carried out using one-way ANOVA followed by Tukey’s multiple comparisons test by the GraphPad Prism software (version 8). The level of significance was set at *P* < 0.05 for all performed analysis.

## Results

### DAPA mitigated neurobehavioral changes in OVX/d-Gal rats

**Fig. 2:**
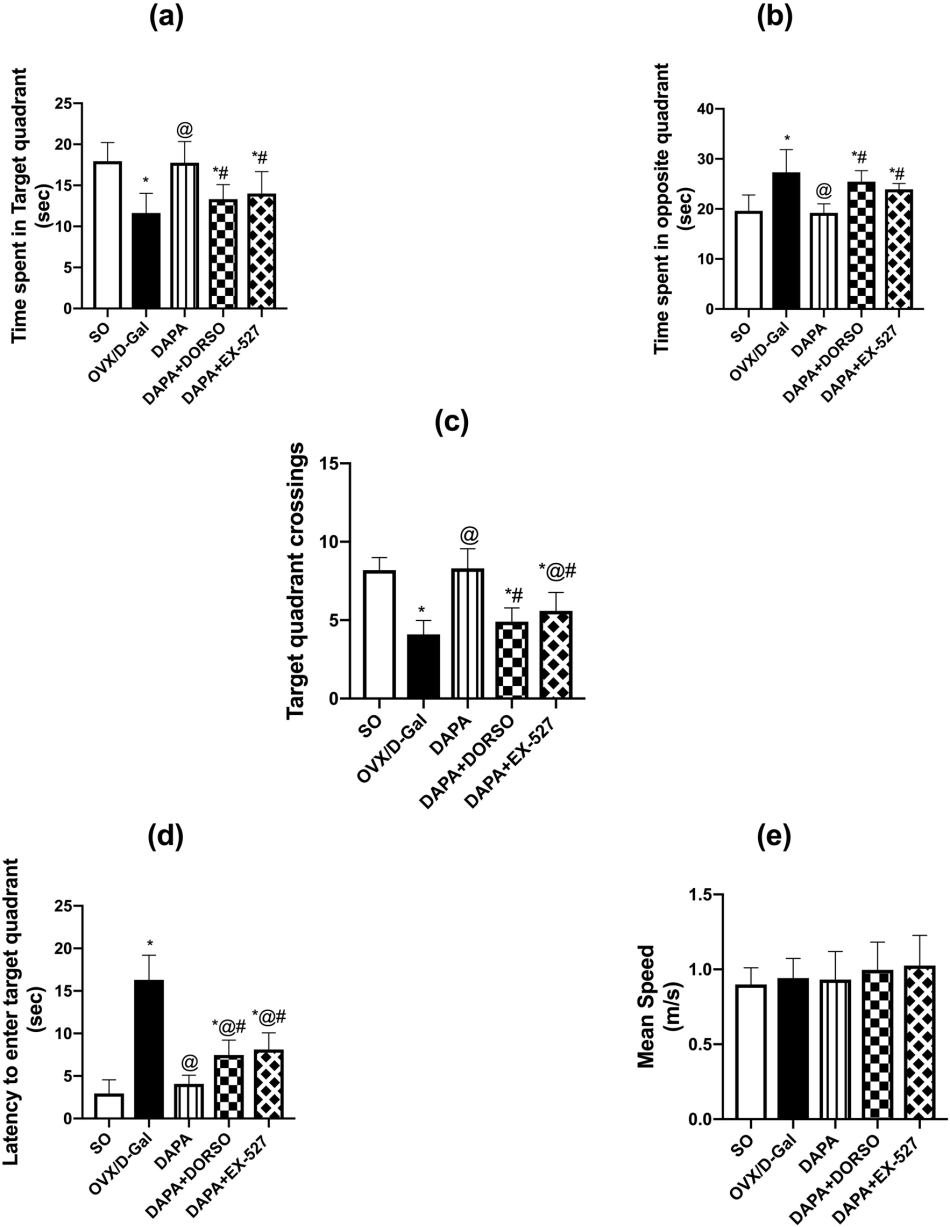
Effect of DAPA alone or in combination with DORSO or EX-527 on OVX/D-Gal-induced memory dysfunction in Morris water maze test. **a** Time spent in target quadrant, **b** time spent in opposite quadrant, **c** target quadrant crossings, **d** latency to enter target quadrant, and **(e)** mean speed. Data were expressed as mean ± SD (*n* = 10). *vs. SO, ^@^vs. OVX/D-Gal, ^#^vs. DAPA (one-way ANOVA followed by Tukey’s post hoc test, *P* < 0.05)

Rats were examined in MWM task to assess their cognitive abilities as verified by time in target quadrant (F _(4, 45)_ = 14.04; *P* < 0.0001), time in opposite quadrant (F _(4, 45)_ = 16.16; *P* < 0.0001), escape latency (F _(4, 45)_ = 72.74; *P* < 0.0001), and frequency (F _(4, 45)_ = 36.44; *P* < 0.0001). OVX/d-Gal group spent significantly less time in the target quadrant (*P* < 0.0001; 35%) and halved the crossings (*P* < 0.0001) to navigate for the missed platform in the target quadrant as compared to SO rats along with retarded entrance to target quadrant (*P* < 0.0001; 5.5-fold), while increased the searching time for the escape platform in the opposite quadrant (*P* < 0.0001; 39%). Treatment with DAPA significantly retrieved the memory functions as evidenced by an increase in time spent in the target quadrant and crossings (*P* < 0.0001; 1.5- and twofold, respectively), while lowered the latency to enter the target quadrant and their navigation in the opposite quadrant (*P* < 0.0001; 75 and 30%, respectively) compared to untreated rats. On the other hand, DORSO- or EX-527-administered rats dampened DAPA’s memory ameliorating effect as indicated by the delayed entrance to target quadrant by 1.8- (*P* = 0.003) and twofold (*P* = 0.0003), and the increased direction to opposite quadrant by 1.3- (*P* = 0.0001) and 1.2-fold (*P* = 0.0046), respectively, while decreasing the time in target quadrant by 25 (*P* = 0.0011) and 21% (*P* = 0.0082) as well as the crossings by 41 (*P* < 0.0001) and 33% (*P* < 0.0001), respectively, when compared to the DAPA-treated rats. There was no significant difference detected between the experimental groups in the mean speed (F _(4, 45)_ = 0.932; *P* = 0.4536) (Fig. 2).

### DAPA alleviated histopathological changes in OVX/d-Gal rats

**Fig. 3:**
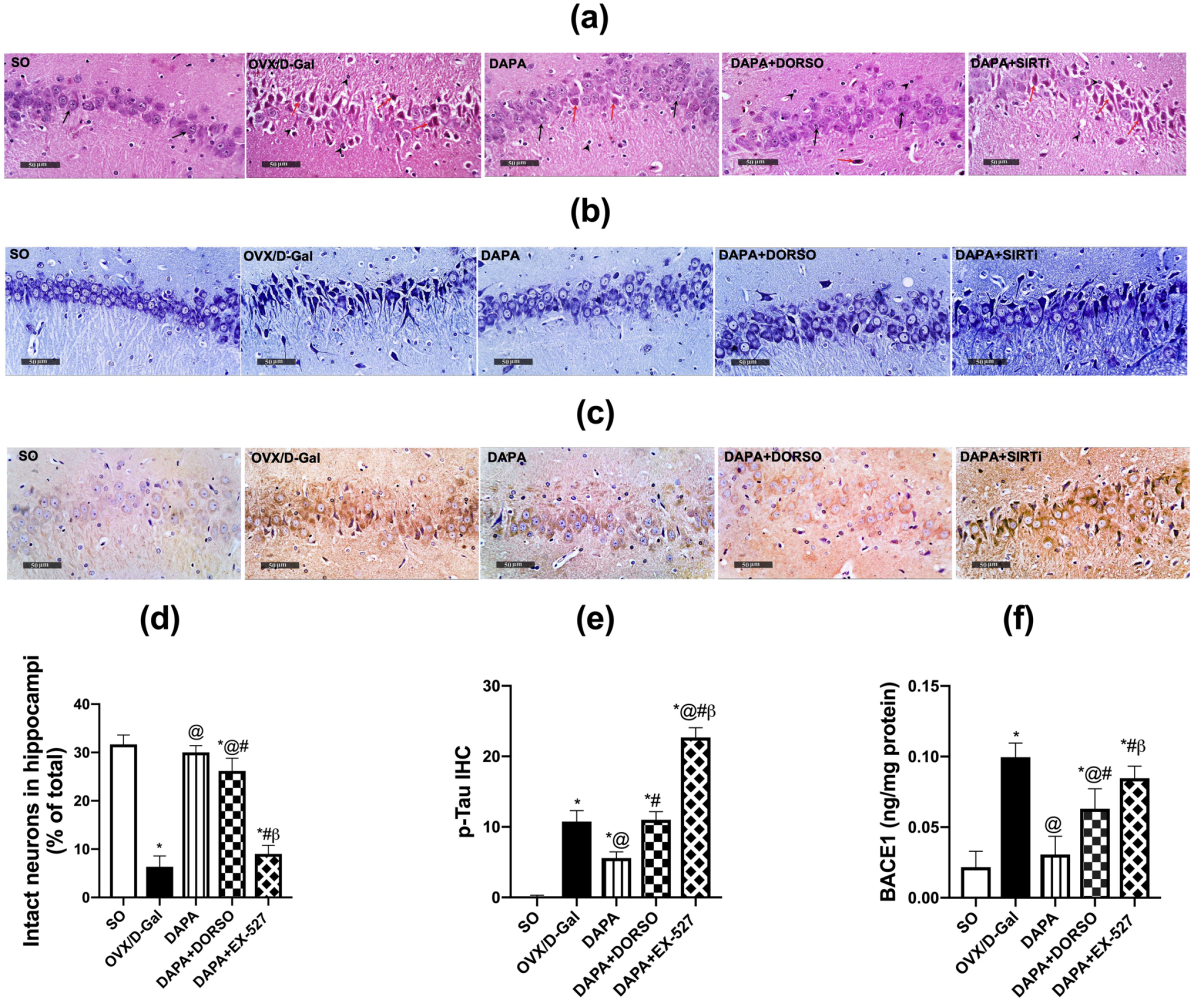
Effect of DAPA alone or in combination with DORSO or EX-527 on OVX/D-Gal-induced histopathological alterations as well as AD hallmarks (p-Tau and BACE1). **a** Representative H&E photomicrographs, **b** illustrative Nissl-stained photomicrographs, and **c** photomicrographs of p-Tau immuno-histochemical expression. Magnifications: ×400. Bar charts representing **d** intact neurons and **e** p-Tau positive cells as well as **f** BACE1 content in hippocampi of each group where each bar with vertical line illustrating the mean ± SD (*n* = 3–6). *vs. SO, ^@^vs. OVX/D-Gal, ^#^vs. DAPA, ^β^vs. DAPA + DORSO (one-way ANOVA followed by Tukey’s post hoc test, *P* < 0.05)

ANOVA showed significant effect on the histopathological changes (F _(4, 10)_ = 182.3; *P* < 0.0001). Normal rats demonstrated normal morphological features for the hippocampal layers with obvious intact well-organized pyramidal neurons; showing intact subcellular details and intercellular matrix along with minimal glial cells infiltrates. Ovariectomized rats injected with d-Gal showed severe neuronal damage and loss of intact cell count manifested in Nissl stain (*P* < 0.0001; 81%) when compared with normal rats, with abundant figures of hyper-eosinophilic, shrunken, and pyknotic neuronal cell bodies losing its subcellular details, accompanied with severe perineuronal edema and moderate increase of reactive glial cells infiltrates. Rats treated with DAPA showed significant neuroprotective efficacy with almost well-organized histological features of hippocampal layers with many apparent intact neurons supported by increased Nissl stain (*P* < 0.0001; 4.9-fold) when compared to OVX/d-Gal rats, in addition to minimal sporadic records of degenerated and necrotic pyramidal neurons. Moreover, a mild persistence of glial cells infiltrates as well as minimal edema of brain matrix were observed. Rats treated with DORSO concurrently with DAPA presented milder severity of neuronal damage and loss supported by more records of apparent intact neurons in Nissl stain micrographs (*P* < 0.0089; 18%) compared with DAPA group rats, additionally, mild glial cells infiltrates were observed. Inhibition of SIRT1 by EX-527 demonstrated almost the same presentation of OVX/d-Gal animals and impeded the protective effect of DAPA causing a decrease in intact cells’ count by 67% (*P* < 0.0001) when compared to DAPA-treated rats. Moreover, EX-527-treated rats displayed a decline in the count of intact neurons in Nissl staining (*P* < 0.0001; 59%) compared to DORSO-treated rats (Fig. 3).

### DAPA lowered the hallmarks of Alzheimer’s disease (BACE1, p-Tau) in OVX/**d**-Gal rats

BACE1 and p-Tau are considered one of the major contributors to AD. By the same token, ANOVA showed significant differences among groups; (F _(4, 25)_ = 51.08; *P* < 0.0001) for BACE1 and (F _(4, 10)_ = 547.5; *P* < 0.0001) for p-Tau. Administration of d-Gal to estrogen-deprived rats enhanced the hippocampal BACE1 content and p-Tau immuno-histochemical expression by 4.6- and 120-fold (*P* < 0.0001), respectively, as compared to SO group rats. Daily administration of DAPA mitigated OVX/ d-Gal-induced elevation in AD hallmarks to 31% (BACE1; *P* < 0.0001) and 41% (p-Tau; *P* < 0.0001). Inhibition of AMPK and SIRT1 by DORSO and EX-527, respectively, increased significantly BACE1 to 2.1-fold (*P* = 0.0005) and 2.8-fold (*P* < 0.0001), respectively, together with enhanced the phosphorylation of Tau to 2.4- and 4.9-fold (*P* < 0.0001), respectively, as compared to DAPA-treated rats. The administration of EX-527 showed more significant increase in Tau phosphorylation (*P* < 0.0001; twofold) and BACE1 (*P* = 0.0249; 1.3-fold), respectively, as related to DORSO-treated rats (Fig. 3).

### DAPA enhanced the energy sensors (AMP/ATP, LKB1, AMPKα_1,2_) in OVX/d-Gal rats

**Fig. 4:**
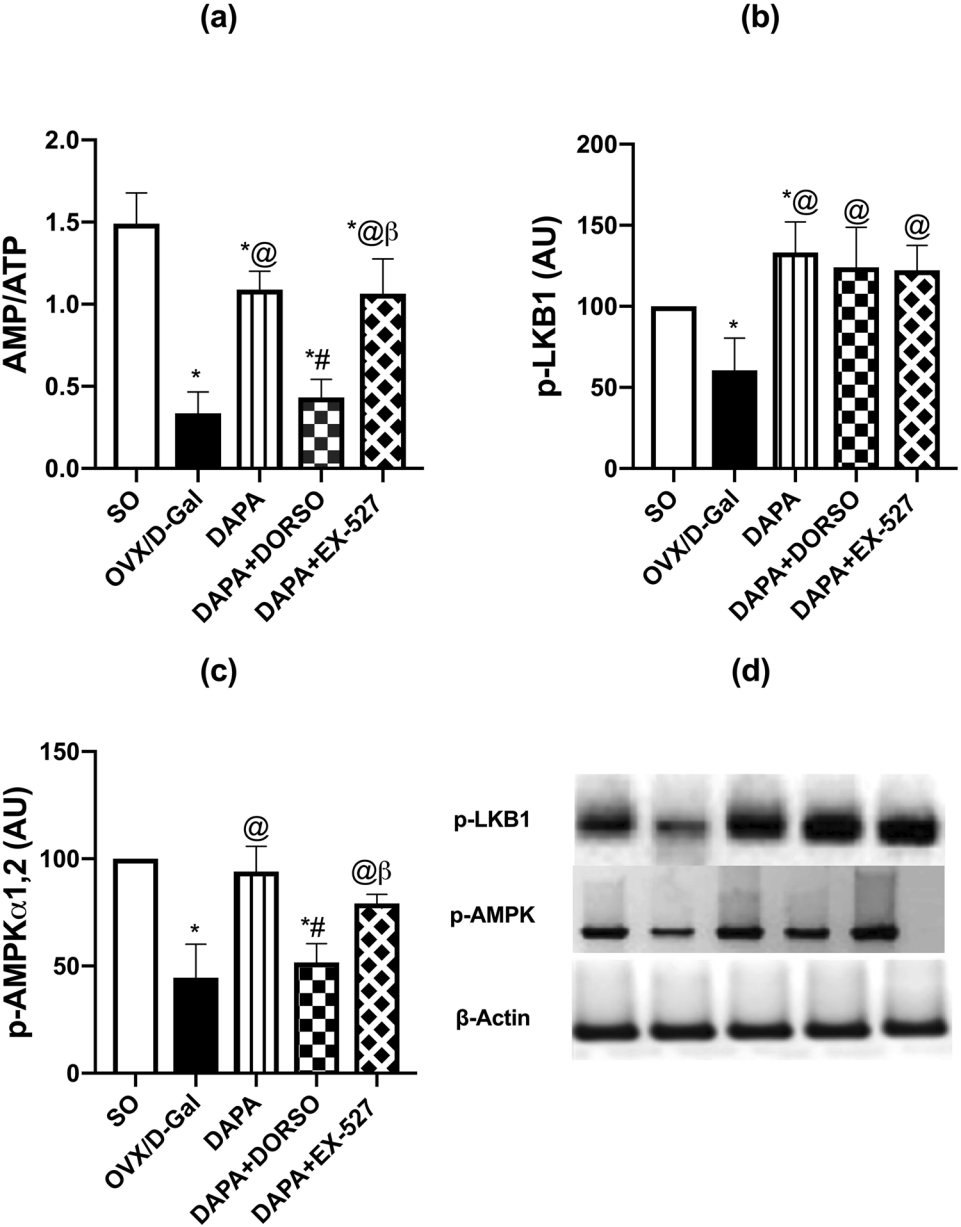
Effect of DAPA alone or in combination with DORSO or EX-527 on OVX/D-Gal-induced changes in **a** AMP/ATP ratio and protein expressions of **b** p-LKB1 and **c** p-AMPKα1,2 in the hippocampus as well as **d** western blots of p-LKB1 and p-AMPKα1,2. Data were expressed as mean ± SD (*n* = 3–6). *vs. SO, ^@^vs. OVX/D-Gal, ^#^vs. DAPA, ^β^vs. DAPA + DORSO (one-way ANOVA followed by Tukey’s post hoc test, *P* < 0.05)

Disturbance in cellular energy is crucial in AD progression. Hippocampal expression of energy sensors showed significant differences among groups; AMP/ATP (F _(4, 25)_ = 58.77; *P* < 0.0001), LKB1 (F _(4, 15)_ = 10.67; *P* = 0.0003), and AMPK (F _(4, 15)_ = 25.86; *P* < 0.0001). Removal of ovaries with d-Gal administration lowered AMP/ATP ratio by 77% (*P* < 0.0001) in comparison with SO rats. DAPA reversed the ratio by 3.2-fold of untreated one (*P* < 0.0001). DORSO co-administration hindered DAPA’s effect to 40% (*P* < 0.0001), while EX-527 did not elicit any significant effect on DAPA’s outcome. Protein expression of p-LKB1 and its downstream mediator, p-AMPKα1,2 was dampened by 40 (*P* = 0.0469) and 56% (*P* < 0.0001), respectively, in OVX/d-Gal group as compared with SO group. DAPA counteracted the OVX/d-Gal-induced depression in p-LKB1 and p-AMPKα1,2 protein expression to reach 2.2- (*P* = 0.0003) and 2.1-fold (*P* < 0.0001), respectively. On the other hand, DAPA-induced up-leveling of p-AMPKα1,2 was hampered upon concurrent administration of DORSO to 55% (*P* = 0.0002) but was unaffected by EX-527 co-administration. Regarding DAPA’s up-leveling effect on LKB1, it was not affected by either AMPK or SIRT1 inhibition (Fig. 4).

### DAPA regulated autophagy pathway and mitochondrial function (SIRT1, mTOR, Beclin1, LC3B and TFAM) in OVX/d-Gal rats

**Fig. 5:**
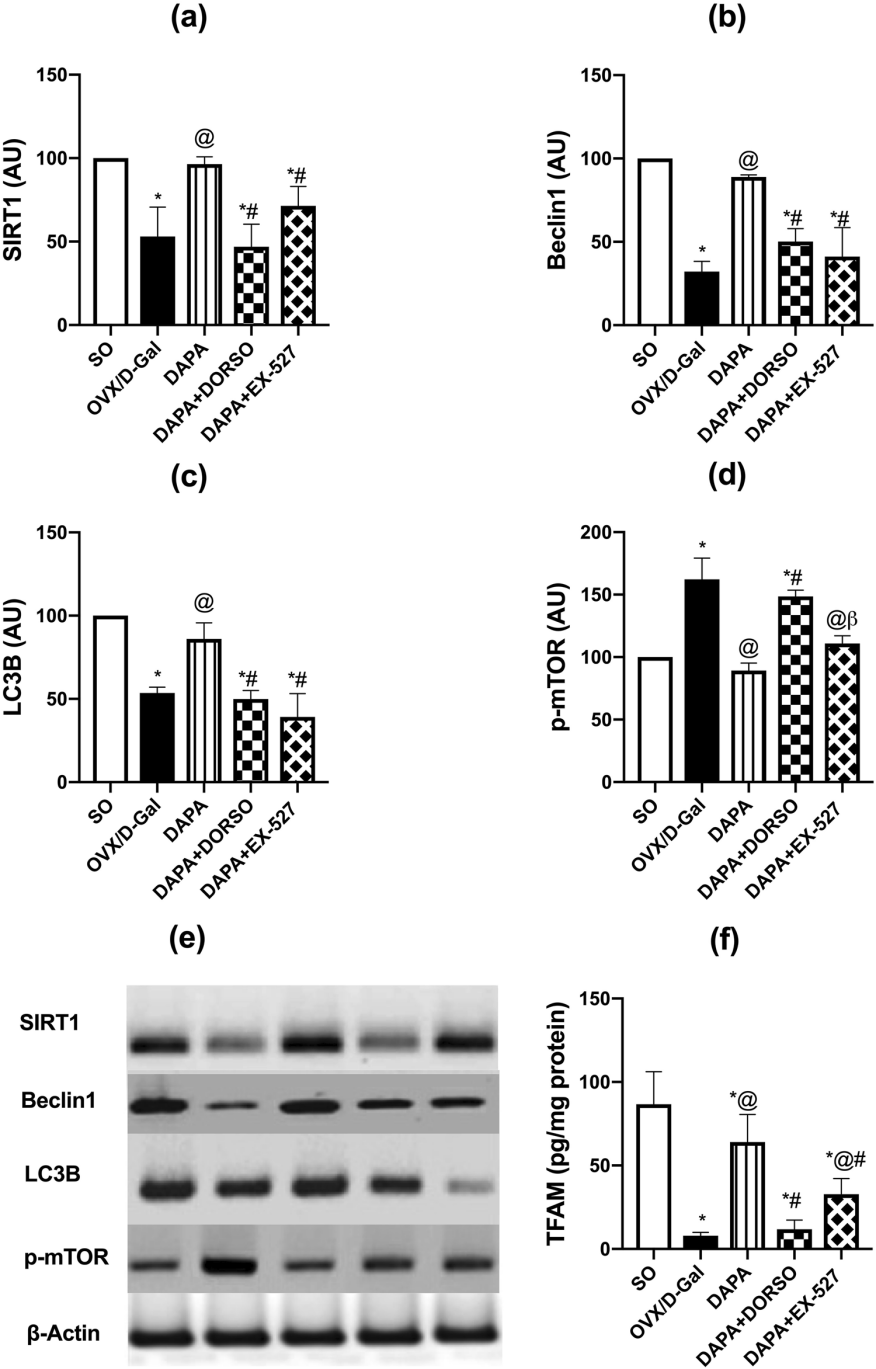
Effect of DAPA alone or in combination with DORSO or EX-527 on OVX/D-Gal-induced changes in the expression of **a** SIRT1, **b** Beclin1, **c** LC3B, **d** p-mTOR proteins in the hippocampus, and **e** corresponding western blots as well as **f** TFAM hippocampal content. Data were expressed as mean ± SD (*n* = 3–6). *vs. SO, ^@^vs. OVX/D-Gal, ^#^vs. DAPA, ^β^vs. DAPA + DORSO (one-way ANOVA followed by Tukey’s post hoc test, *P* < 0.05)

Dysfunction in autophagic pathways with mitochondria is common in AD as demonstrated by the hippocampal levels of SIRT1 (F _(4, 15)_ = 18.13; *P* < 0.0001), mTOR (F _(4, 10)_ = 38.15; *P* < 0.0001), Beclin1 (F _(4, 10)_ = 34.15; *P* < 0.0001), LC3B (F _(4, 10)_ = 30.78; *P* < 0.0001), and TFAM (F _(4, 25)_ = 44.93; *P* < 0.0001). d-Gal injection in OVX rats evoked prominent decrease in protein expression of SIRT1, LC3B, and Beclin1 to 53% (*P* = 0.0003), 54% (*P* = 0.0003), and 32% (*P* < 0.0001), respectively, while elevating that of mTOR by 1.6-fold (*P* < 0.0001) as compared with SO group rats (Fig. 5). DAPA counteracted OVX/d-Gal-induced changes in the protein expression of the previously mentioned parameters by 1.8-fold (*P* = 0.0006), 1.6-fold (*P* = 0.0042), and 2.8-fold (*P* = 0.0001), as well as 45% (*P* < 0.0001), respectively. Administration of DORSO along with DAPA lowered the protein expressions of SIRT1, LC3B, and Beclin1 by 51% (*P* = 0.0002), 42% (*P* = 0.002), and 43% (*P* = 0.0025), respectively, while increasing that of mTOR by 1.7-fold (*P* < 0.0001) in comparison with DAPA group. EX-527 also mitigated DAPA-induced increase in SIRT1, LC3B, and Beclin1 protein levels to 74% (*P* = 0.05), 46% (*P* = 0.0002), and 46% (*P* = 0.0005), respectively, without blocking DAPA’s alleviating effect on mTOR expression. Regarding TFAM level, it was significantly lowered upon d-Gal injection along with ovarian excision by 91% (*P* < 0.0001) of SO group. On the other hand, treatment with DAPA caused a prominent rise in its level to eightfold (*P* < 0.0001) of that of OVX/d-Gal group. DORSO or EX-527 co-administration counteracted TFAM up-leveling afforded by DAPA by 82% (*P* < 0.0001) and 49% (*P* = 0.0017), respectively.

## Discussion

The current study revealed the cognitive enhancing effect of DAPA in a rat model of AD triggered by ovarian removal along with d-Gal injection. This conception is supported by ample lines of evidence: (i) improving spatial memory capabilities and mitigating histopathological alterations, (ii) reducing AD hallmarks (p-Tau and BACE1), (iii) increasing AMP/ATP ratio and p-LKB1 expression which were associated with heightened hippocampal expression of p-AMPK and SIRT1, (iv) modulating autophagic markers; increasing LC3B and Beclin1 while decreasing mTOR, and (v) promoting TFAM expression, a marker for mitochondrial biogenesis. Interestingly, the present work suggested the involvement of LKB1/AMPK/SIRT1-induced autophagy and mitochondrial biogenesis in DAPA neuroprotection against OVX/d-Gal-induced AD-like pathology.

In the present research, OVX/d-Gal group rats exhibited cognitive impairment and memory loss as indicated by their performance in MWM test in accordance with previous findings (Ibrahim et al. 2019, 2020; Kamel et al. 2018). Instead, DAPA-treated group showed spatial memory improvement that reflected the amendment of AD-like histological alterations. In agreement, DAPA attenuated the cognitive impairment induced in rats which were fed on high-fat diet as indicated in MWM test and enhanced their brain function (Sa-nguanmoo et al. 2017). Interestingly, DAPA’s effect was mitigated in the presence of DORSO or EX-527, highlighting the involvement of AMPK/SIRT1 signaling in mitigating the decline in cognition.

Amyloid β-peptide is a molecular culprit in AD which is formed via β- and γ-secretases-mediated cleavage of amyloid precursor protein (APP) and it clumps into plaques between neurons. These plaques motivate the massive neuroinflammation and synaptic dysfunction as well as neuronal death, principally in the hippocampus, resulting in early memory dysfunction (Mondragón-Rodríguez et al. 2010). Cleavage by β-secretase (BACE1) is an important step for Aβ generation, and elevation in BACE1 levels was reported in patients and investigational models of AD (Tamagno et al. 2012). Herein, treatment with DAPA hindered OVX/d-Gal-triggered increase in hippocampal BACE1. In context, empagliflozin was reported to reduce Aβ, Tau phosphorylation and brain atrophy along with improving learning and memory in a mixed mice model of AD and diabetes type 2 (Hierro-Bujalance et al. 2020).

In AD, the microtubule-bound Tau protein is subjected to abnormal hyperphosphorylation and then accumulated together forming neurofibrillary tangles inside the neurons (Medina and Avila 2014). These tangles block the axonal transport system which impairs synaptic communication, leading to neuronal death. Herein, a substantial increase of Tau phosphorylation was detected in OVX/d-Gal rats, whereas treatment with DAPA curbed such hyperphosphorylation. Interestingly, DAPA-induced mitigation of BACE1 and p-Tau was counteracted by AMPK or SIRT1 inhibition, indicating AMPK/SIRT1 signaling involvement in DAPA’s neuroprotective effect against AD-associated anomalies.

In the current study, DAPA enhanced hippocampal p-AMPKα1,2 expression in OVX/d-Gal-subjected rats. It was formerly reported that activation of AMPK in patients suffering from neurodegenerative disease can ameliorate energy metabolism and protein clearance in their brains (Bayliss et al. 2016; Ashabi et al. 2014). In addition, activation of AMPK hampered dopaminergic neurotoxicity in an experimental model of Parkinson’s disease (Lu et al. 2016). Moreover, AMPK phosphorylation effectively delayed brain aging and the resultant dementia in the senescence accelerated mouse-8 model of aging, by improving autophagy and mitochondrial function (Xu et al. 2020). However, existing studies that put insights to the relation of AMPK and SGLT2 inhibitors are few. For instance, canagliflozin was reported to cause AMPK activation in HEK-293 cells and hepatocytes (Hawley et al. 2016). It was also suggested that the inhibitory effect of DAPA on myocardial inflammation and apoptosis as well as the progression of diabetic cardiomyopathy in type 2 diabetic mice was related to AMPK activation (Ye et al. 2017). Thus, AMPK activation is a suggested molecular mechanism in DAPA-induced cognitive enhancing effect.

Herein, treatment with DAPA led to a prompt elevation in AMP/ATP ratio as related to OVX/d-Gal group rats. The increase in intracellular AMP levels activating the nature’s energy sensor, AMPK. Binding of AMP allosterically activates AMPK up to tenfold, additionally, AMP increases AMPK phosphorylation at Thr 172 and protect against its dephosphorylation (Ross et al. 2016). The effects of AMP are counteracted by ATP, thus the regulation of AMPK relies on AMP/ATP ratio (Hardie et al. 1999).

LKB1 is the upstream activating factor of AMPK. Herein, DAPA administration abrogated OVX/d-Gal-induced depression in LKB1 expression causing a significant rise in its level. Furthermore, phosphorylation/activation of LKB1 was compatible with that of AMPK; where the increased expression of LKB1 protein prompted by DAPA was accompanied by a significant rise in AMPK expression. Moreover, LKB1 was still prominently activated when DORSO and EX-527 inhibited the activation of AMPK and SIRT1, respectively, indicating that DAPA can activate AMPK via its upstream regulator LKB1. In context, it has been stated that LKB1 phosphorylation at Ser428 residue enhances its capability to bind and activate AMPK (Xie et al. 2006).

The current study investigated whether the enhancing effect of DAPA on LKB1 expression is SIRT1-dependent. A former investigation reported that SIRT1 can induce LKB1 deacetylation as well as its phosphorylation (Wang et al. 2018). However, the current study revealed that LKB1 expression stimulated by DAPA was not abrogated by SIRT1 inhibition, proposing that DAPA could phosphorylate LKB1 directly independent of SIRT1.

The present results showed that DAPA administration significantly increased SIRT1 expression and this effect was blocked with either AMPK or SIRT1 inhibitors as evidenced by a prompt decrease in its expression. SIRT1 is a main downstream molecule in AMPK signaling, that exerts neuroprotective effects (Li and Han 2018; Tulino et al. 2016). SIRT1 exerts an imperative role in suppressing AD pathology by direct and indirect mechanisms. It stimulates α-secretases and non-amyloidogenic processing of APP, and consequently reducing Aβ levels (Qin et al. 2006). Also, it suppresses the induction of microglial proliferation and its mediated neuroinflammation (Li and Han 2018; Cho et al. 2015). Additionally, SIRT1 enhances autophagic removal of Aβ and p-Tau, and it promotes the deacetylation of Tau triggering its degradation (Min et al. 2010). Furthermore, SIRT1 suppresses neuronal apoptosis by modulating the activity of tumor suppressor protein, p53 (Xiong et al. 2015). The level of SIRT1 was decreased in AD patients’ brains, particularly in the brain regions that are influenced during the progress of the disease, in correlation with increased Tau levels (Lutz et al. 2014). Mounting evidence has documented that the amelioration of AD-associated pathology induced by various compounds is correlated with SIRT1 elevation (Sun et al. 2014; Marwarha et al. 1842; Lee et al. 2014). Moreover, it has been reported that overexpression of AMPKα1 ameliorated post-operative cognitive dysfunction through AMPK/SIRT1 and autophagy pathway (Yan et al. 2019). AMPK is a potent activator of SIRT1 via modulating the cellular NAD^+^/NADH ratio (Cantó et al. 2009). Accordingly, the present results suggested that AMPK-mediated activation of SIRT1 signaling may be an imperative mechanism involved in DAPA’s effect to halt AD pathology in OVX/d-Gal rats.

Defective autophagy is closely related to neurodegenerative diseases (Nixon 2013; Bergamini 2006). Whereas, promoted autophagy can effectively mitigate cognitive dysfunction and protect neuronal cells against cellular insults (Song et al. 2017; Dai et al. 2017; Dong et al. 2015). Thus, autophagy is suggested to be an efficient strategy to ameliorate neurodegeneration. LC3B and Beclin1 are proteins involved in autophagy regulation. Additionally, mTOR is a key blocker of autophagosome formation, thus exerts a pathogenic impact in AD via inhibiting the autophagic removal of Aβ and p-Tau aggregates (Querfurth and Lee 2021). The present study showed that OVX and d-Gal administration produced a substantial reduction in hippocampal expression of LC3B and Beclin1, along with up-leveling of mTOR. Such effect was obviously reversed by DAPA treatment. Interestingly, AMPK inhibition by DORSO attenuated DAPA’s effect on the autophagy markers resulting in reduced LC3B and Beclin1 expression along with elevated mTOR signaling. On the other hand, SIRT1 inhibition by EX-527 abrogated DAPA-mediated elevation of LC3B and Beclin1 expression without affecting the drug’s inhibitory effect on mTOR. These results proposed that mTOR suppression afforded by DAPA might be attributed to AMPK activation independently on SIRT1 involvement, whereas activation of AMPK and SIRT1 could participate together in LC3B and Beclin1 up-leveling. Indeed, AMPK activation can enhance autophagy through multiple signaling pathways (Jiang et al. 2015). It has been reported that AMPKα1 overexpression ameliorated post-operative cognitive dysfunction in rats through the activation of the hippocampal autophagy signals as indicated by increasing LC3B and Beclin1 and reducing p62 expression along with upregulating p‐AMPK and SIRT1; these effects were significantly attenuated by DORSO administration (Yan et al. 2019). Moreover, AMPK is a negative regulator of mTOR activation through phosphorylating tuberous sclerosis complex 2, a component of the mTOR inhibitory complex (Alers et al. 2012). In addition, resveratrol activated neuronal autophagy and consequently increased Aβ clearance in a transgenic AD mouse model via activating AMPK with subsequent mTOR inhibition (Vingtdeux et al. 2010). Therefore, the present results suggested that the autophagic enhancing effect of DAPA mediated by activating AMPK and/or SIRT1 may be a potential mechanism involved in its capability to subside AD pathological aberrations in OVX/d-Gal rats.

Preserving mitochondrial function is essential for the treatment of neurodegenerative disorders (Li et al. 2017). TFAM is considered as an ultimate marker for mitochondrial biogenesis. In the current work, OVX/d-Gal rats displayed a pronounced decline in hippocampal TFAM, reflecting the dysregulated mitochondrial function. Mitochondrial biogenesis in hippocampal neurons was found to be impaired in Aβ-treated hippocampal cell culture (Dong et al. 2016) as well as in AD patients and animal models (Sheng et al. 2012; Rice et al. 2014). Aβ gradually builds up in the mitochondria (Manczak et al. 2011), stops its DNA replication and hinders the mitochondrial biogenesis process (Dong et al. 2016). This ultimately leads to neuronal metabolic deficiency, reactive species generation, and neuronal death (Smith et al. 2017), resulting in memory decline and cognitive dysfunction. Thus, activation of mitochondrial biogenesis may assist in limiting AD progression. Herein, DAPA treatment mitigated OVX/d-Gal’s effect causing a significant increase in TFAM expression, and this effect was abolished with AMPK or SIRT1 inhibition. In context, melatonin-induced neuroprotective effect in Aβ-injected rats was mediated through mitochondrial biogenesis following SIRT1 signaling activation (Ansari Dezfouli et al. 2019). SIRT1 deacetylates and activates peroxisome proliferator-activated receptor-γ coactivator-1α, which is the principal transcriptional cofactor for mitochondrial biogenesis (Wang et al. 2015). Thus, enhanced mitochondrial biogenesis mediated through AMPK/SIRT1 signaling may be implicated in DAPA’s effect against AD.

## Conclusion

The present results propose DAPA as a promising autophagy-enhancing therapy for AD. DAPA improved spatial memory, mitigated AD-associated histopathological alterations, and reduced p-Tau and BACE1 in the OVX/d-Gal rat model of AD. It also increased the energy sensors AMP/ATP, LKB1, and AMPK. It modified autophagy-related proteins where it enhanced LC3B and Beclin1 while reduced mTOR. Finally, DAPA enhanced mitochondrial biogenesis as TFAM expression was increased. Noteworthy, this study proposed LKB1/AMPK/SIRT1 signaling as a contributor to the anti-AD effect of DAPA.

## Data Availability

The datasets generated during and/or analyzed during the current study are available from the corresponding author on reasonable request.
